# A Social Media Website (Supporting Our Valued Adolescents) to Support Treatment Uptake for Adolescents With Depression or Anxiety: Pilot Randomized Controlled Trial

**DOI:** 10.2196/35313

**Published:** 2022-10-07

**Authors:** Ana Radovic, Yaming Li, Doug Landsittel, Kayla R Odenthal, Bradley D Stein, Elizabeth Miller

**Affiliations:** 1 Division of Adolescent and Young Adult Medicine University of Pittsburgh Medical Center Children’s Hospital of Pittsburgh University of Pittsburgh School of Medicine Pittsburgh, PA United States; 2 Department of Biomedical Informatics University of Pittsburgh Pittsburgh, PA United States; 3 Department of Epidemiology and Biostatistics Indiana University School of Public Health Bloomington, IN United States; 4 RAND Corporation Pittsburgh, PA United States

**Keywords:** adolescent, adolescent health services, technology, depression, anxiety

## Abstract

**Background:**

Adolescents with depression or anxiety initiate mental health treatment in low numbers. Supporting Our Valued Adolescents (SOVA) is a peer support website intervention for adolescents seen in primary care settings and their parents with the goal of increasing treatment uptake through changing negative health beliefs, enhancing knowledge, offering peer emotional support, and increasing parent-adolescent communication about mental health.

**Objective:**

This pilot study aimed to refine recruitment and retention strategies, refine document intervention fidelity, and explore changes in study outcomes (the primary outcome being treatment uptake).

**Methods:**

We conducted a 2-group, single-blind, pilot randomized controlled trial in a single adolescent medicine clinic. Participants were aged 12 to 19 years with clinician-identified symptoms of depression or anxiety for which a health care provider recommended treatment. The patient and parent, if interested, were randomized to receive the SOVA websites and enhanced usual care (EUC) compared with EUC alone. Baseline, 6-week, and 3-month measures were collected using a web-based self-report survey and blinded electronic health record review. The main pilot outcomes assessed were the feasibility of recruitment and retention strategies. Implementation outcomes, intervention fidelity, missingness, and adequacy of safety protocols were documented. Descriptive statistics were used to summarize mental health service use and target measures with 2-sample *t* tests to compare differences between arms.

**Results:**

Less than half of the adolescents who were offered patient education material (195/461, 42.2%) were referred by their clinician to the study. Of 146 adolescents meeting the inclusion criteria, 38 completed the baseline survey, qualifying them for randomization, and 25 (66%, 95% CI 51%-81%) completed the 6-week measures. There was limited engagement in the treatment arm, with 45% (5/11) of adolescents who completed 6-week measures reporting accessing SOVA, and most of those who did not access cited forgetting as the reason. Changes were found in target factors at 6 weeks but not in per-protocol analyses. At 12 weeks, 83% (15/18) of adolescents randomized to SOVA received mental health treatment as compared with 50% (10/20) of adolescents randomized to EUC (*P*=.03).

**Conclusions:**

In this pilot trial of a peer support website intervention for adolescents with depression or anxiety, we found lower-than-expected study enrollment after recruitment. Although generalizability may be enhanced by not requiring parental permission for adolescent participation in the trials of mental health interventions, this may limit study recruitment and retention. We found that implementing education introducing the study into provider workflow was feasible and acceptable, resulting in almost 500 study referrals. Finally, although not the primary outcome, we found a signal for greater uptake of mental health treatment in the arm using the SOVA intervention than in the usual care arm.

**Trial Registration:**

ClinicalTrials.gov NCT03318666; https://clinicaltrials.gov/ct2/show/NCT03318666

**International Registered Report Identifier (IRRID):**

RR2-10.2196/12117

## Introduction

### Background

Adolescent depression and anxiety are rapidly increasing public health problems in the United States, with serious clinical and societal consequences [1-3]. One-third of adolescents with depression experience suicidality and 11% attempt suicide [4], resulting in US $12 billion in hospital costs [5], but only one-third receive treatment [6]. An estimated 30% to 70% of children with mental health disorders do not receive counseling from a mental health professional [7]. Routine adolescent depression screening has been widely implemented in pediatric primary care settings to increase the identification of depression in early adolescence [8,9]; however, initiation of treatment is low with screening without subsequent efforts to increase engagement [10-12]. There is a need for feasible and scalable additional interventions that can be implemented in busy primary care settings, which can accompany screening and address attitudinal factors in both parents and adolescents, which may enhance treatment engagement.

Supporting Our Valued Adolescents (SOVA) [13] is a moderated website (with an accompanying separate parent website, wiseSOVA) [14] with the overall goal of increasing the uptake of and engagement with mental health treatment in adolescents referred to treatment. SOVA is designed to target (1) increasing mental health literacy, (2) prevailing over negative health beliefs toward depression or anxiety diagnosis and treatment, and (3) growing an anonymous online support community. The conceptual model and rationale for the design of the SOVA intervention are described in a previous protocol paper [15]. The SOVA Peer Ambassador program scaffolds websites by engaging youths (age 14-21 years) who have already experienced symptoms of depression or anxiety, mostly those who have already been in treatment and are willing to write monthly blog posts and comment weekly on others’ posts to share their experiences with mental health, with the goal of encouraging others to find support. One-third of the posts are written by SOVA Peer Ambassadors, and two-thirds, by the research team.

### Aims and Objectives

The aims of this pilot randomized controlled trial of SOVA as compared with enhanced usual care (EUC) were as follows:

Aim 1: examine the feasibility of and refine recruitment and retention strategies with the goal of recruiting 150 adolescents with 75 per arm and that 90% of the sample would complete 6-week follow-up measures.

Aim 2: document implementation outcomes and intervention fidelity.

Exploratory aim 3: describe changes in and between-arm comparisons for the following:

1. Health beliefs and knowledge
2. Emotional and informational social support
3. Adolescent and parent communication quality
4. Perceived need for treatment
5. Mental health service use
6. Symptoms of depression and anxiety

Aim 4: examine the appropriateness of effectiveness and implementation measures, rates of missing data, and adequacy of safety protocols.

## Methods

A detailed description of the protocol and design of this study is available in a prior publication [15].

### Trial Design

This was a parallel-arm, single-blind, pilot randomized controlled trial with 1:1 allocation and 6-week and 12-week follow-up by self-report and electronic health record (EHR) review.

### Participants

The trial participants included adolescents and their parents, if interested. Adolescent health care providers (AHCPs) included medical providers (physicians and advanced practice practitioners) seeing adolescent patients for physical health visits and were interviewed at study conclusion. Adolescents were eligible if they were between the ages of 12 and 19 years inclusive, their AHCP identified that they have symptoms of anxiety or depression, and the AHCP recommended that the adolescent initiate a new treatment episode (no treatment in the past 3 months). Adolescents were excluded if they were already engaged in treatment, meaning they were currently on a psychiatric medication—defined as filling a prescription and starting to and currently taking a medication—or currently engaged in psychotherapy—defined as attending at least 3 therapy sessions in the past 2 months—for depression or anxiety. The purpose of this was to measure specifically treatment initiation in adolescents not currently in treatment, as the goal of SOVA is to serve as an intervention that promotes psychotherapy treatment initiation and engagement. Adolescents were not excluded if they were newly being prescribed a medication or referred to psychotherapy at the AHCP visit. Adolescents were required to have at least mild depressive symptoms (≥5 on the Patient Health Questionnaire-9 [PHQ-9] screener [16]) or mild anxiety symptoms (≥5 on the Generalized Anxiety Disorder Questionnaire-7 [GAD-7] screener [17]). Adolescents were excluded if they were actively suicidal, requiring crisis intervention or hospitalization, defined as current suicidal thoughts and plans or AHCP determination. Adolescents were excluded if they had no access to the internet, no active email account, could not read and write in English, or had not completed sixth grade. Initially, adolescents aged 14 to 19 years were recruited because of concerns about a waiver of parental permission but given the minimal risk of the study and the goal to increase earlier engagement in mental health treatment, the age range was expanded to 12 to 19 years.

Parents were included if their child met the study criteria and agreed to enroll in the study, could read and write in English, and had completed sixth grade. Parents were excluded if they had no access to the internet or no active email account. At the start of the study, we initially required adolescents and parents to enroll as a dyad, but as clinical recruitment proceeded, we learned that some adolescents refused to participate in the study because of this requirement, as they were uncomfortable discussing their mental health symptoms with their parents. As the aim of the intervention is partly to enhance communication between an adolescent and their support person, we amended our protocol to not require parents to enroll in the study and were granted a waiver of parental permission. We encouraged adolescents randomized to the SOVA intervention to share the parent website (wiseSOVA) containing publicly accessible articles with a support person, even if the support person was not enrolled in the study. This change occurred after the initial 17 dyads were consented.

AHCPs were included if they were a health care provider (eg, physician, nurse practitioner, or physician assistant) providing clinical services at the 2 participating clinics.

### Procedure

Potential participants were identified by within-clinic research assistants (RAs) reviewing the EHR to determine those who may meet eligibility criteria and approaching patients during clinic visits, as well as self-referral through research advertisements and contact cards placed in clinic spaces. Participants were recruited from 2 clinics providing care to adolescents at an academic medical center, one being general primary care and another being adolescent primary and subspecialty care. RAs prompted AHCPs and other clinical staff including team social workers to introduce the study to the adolescent. Typically, AHCPs would see patients for medical visits for physical health complaints and involve social workers to assist when a mental health referral is indicated. For potentially eligible adolescents (being referred for an initial treatment episode), AHCPs asked the adolescent’s permission to be approached by a research team member available in the clinic or to be contacted after the appointment. RAs then prescreened the EHR to determine if the eligibility criteria were met (specifically, no prior treatment and a referral for a new treatment episode), and if not, they contacted the AHCP to verify. If the individual met the criteria, the RA contacted them to conduct a screen using the PHQ-9 and GAD-7. AHCPs were notified if a potential adolescent participant scored in the severe range or endorsed suicidality. If the eligibility criteria were met, the RA proceeded to obtain consent. Adolescents provided assent, and if their parents were joining the study, the parents provided consent. Then, the RA emailed the adolescent and parent (if participating) an electronic baseline survey using the secure database REDCap (Research Electronic Data Capture; Vanderbilt University) [18]. Randomization was performed after baseline survey completion to either SOVA+EUC or EUC alone. All participants were sent a self-report web-based survey at 6 and 12 weeks, and the research team obtained EHR data at baseline, 6 weeks, and 12 weeks.

### Ethics Approval

Approval was obtained from the University of Pittsburgh Human Research Protection Office (STUDY19120034), including a waiver of parental permission, a waiver of informed consent to review medical records only for the identification of potential participants, and a waiver to obtain a written signed consent form (ie, verbal consent was obtained without a signature). All the participants were emailed a copy of the consent form. The study was registered with the National Institute of Health trials registry, ClinicalTrials.gov (NCT03318666), before participant enrollment. The office did not request an independent data safety monitoring board because, as an adjunct to treatment intervention, the study was deemed as minimal risk.

### Intervention Arm

Several publications describe the SOVA intervention design [19], initial usability testing [20], and evaluation of the moderation process [21]. Briefly, there are 2 separate websites, SOVA for adolescents and young adults and wiseSOVA for parents. All article content is publicly available, but participating in interactive components (ie, comments and discussion boards) requires logging in. Discussion boards are available for any topic. Every weekday, there are new articles, approximately one-third written by SOVA Peer Ambassadors, or young people receiving small compensation for contributing blog article content. This content is reviewed by the research team, providing guidance around factuality before publication. Adolescents randomized to SOVA (and their parent if included) received a welcome email to the sites with log-in information and a phone call or an in-person appointment from an RA to explain site use rules and assist with any log-in technical difficulties. The site use rules included asking participants to not share identifying information, not meet with other participants, avoid bullying, and take a break if they feel upset by using the site. Site moderation (that all comments are reviewed within 24 hours of being posted and removed if they did not meet ground rules) was reviewed. Even if a parent was not enrolled with their adolescent child, adolescents were provided information about the parent website to provide to a parent or guardian or support person if they wished. Adolescents and enrolled parents received a weekly digest email alerting them to new articles published on the site. An Android-only mobile app version of the site, rendering the website in an app form, was also available to the participants. All adolescents in the intervention arm also received EUC.

### Control Arm

As described in detail in a previous publication [22], individuals in the EUC arm and enrolled parents received an email from the research team containing information from the AHCP’s documentation (eg, instructions to follow-up at a certain time interval with the AHCP or instruction to contact an outside therapist or to schedule a follow-up with the clinic therapist) that the research team obtained from the EHR, along with a list of psychoeducational materials and crisis resources. Psychoeducational materials were a list of website materials from US national organizations’ patient-friendly materials on mental health and help seeking; none were connecting to an online peer support community, and none were specific to addressing negative health beliefs about help seeking (Multimedia Appendix 1). The email also included information on how to contact the AHCP and clinic social worker. The clinic social worker conducted their typical duties of assisting with mental health referrals for patients referred by the AHCP.

### Measures

#### Aim 1: Pilot Outcomes

Measures are described in detail in the research protocol manuscript [15] with measure characteristics and reliability and validity metrics included in the research protocol’s accompanying Multimedia Appendix 1, and they are also summarized here in Table 1.

The main pilot study outcome was the study retention rate, measured by the proportion of enrolled adolescents completing the 6-week survey.

**Table 1:** Measures obtained from adolescents and parents at baseline and 6-week follow-up.

			Measure^a^
**Health beliefs**			
	Stigma	Depression Stigma Scale [23]	
	Beliefs about antidepressants	Resistance to Antidepressant Use Questionnaire—higher score indicates more acceptance of antidepressant use; Antidepressant Meanings Scale [24]	
	Beliefs about psychotherapy	Adolescent: barriers to adolescents seeking help [25]; Parent: Parental Barriers to Help Seeking Scale	
**Mental health knowledge**			
	Depression knowledge	Depression Literacy Questionnaire [26]	
	Anxiety knowledge	Anxiety Literacy Questionnaire [26]	
Peer emotional and informational social support			Emotional or Informational subscale from the Medical Outcomes Study Social Support Survey [27]
Parent-adolescent communication quality			Parent-Adolescent Communication Scale [28]
Perceived need for treatment			Single-item yes or no question about need for mental health service [29]; General-Practice Users Perceived-Need Inventory [30]
**Symptoms**			
	Depressive symptoms	Adolescent: Patient Health Questionnaire-9 [31]	
	Anxiety symptoms	Adolescent: Generalized Anxiety Disorder Questionnaire-7 [17]	
Functioning			Adolescent: Multidimensional Adolescent Functioning Scale [32]; Parent: Columbia Impairment Scale-Parent [33]
Relationship quality			Parent-child connectedness [34]

^a^All measures were asked of both adolescents and parents with regard to the adolescents unless specified in the table.

#### Aim 2: Implementation and Fidelity Outcomes

To understand potential strategies to measure future implementation outcomes such as adoption (use of the provider patient education), we gathered data for the number of adolescent patients seen in the clinic where recruitment was taking place and who received information about the study, as measured by a “Stress and Worry” patient education handout inserted into their end-of-visit depart summary during the study timeframe. The depart summary is paperwork that is routinely provided to all patients when they are leaving after their appointment. This helps to determine the feasibility of using patient education to introduce the study. We calculated the proportion of adolescents provided that information who showed an interest in the study to understand the adoption of the trial from those who provided information about it. We also asked AHCP’s to complete a poststudy survey inquiring about whether the provider used the “Stress and Worry” patient education and if so, to what extent on a 4-point Likert scale (0=not at all, 3=to a very great extent) did they think this patient education was clinically useful, intuitively appealing, made sense, used because it was required, and thought colleagues were happy using it and to what extent patients or the social worker brought up the “Stress and Worry” study to the AHCP. AHCPs were also asked open-ended questions about how the study could be improved.

#### Aim 3: Changes in Clinical Outcomes

The proposed main clinical outcome was the use of mental health services. As one of the pilot trial’s goals was to inform measurement selection, mental health service use was measured in multiple ways, per a single-item question combined with EHR data extraction [11], as well as measured by the Actual Help Seeking Questionnaire [35]. Mental health service use was dichotomized into treatment received or not received and assessed at the 6- and 12-week follow-up. Treatment was defined as received if any of the following were true: (1) the adolescent reported yes when asked, “Have you received any treatment for depression or anxiety since the start of this study (this could include starting a new medication, seeing a professional to talk to, or follow up with your adolescent health care provider to talk about depression or anxiety)?”; (2) the adolescent’s parent, if enrolled, reported yes to that same question; or (3) the EHR showed evidence that a new antidepressant medication prescription was filled, an appointment with a mental health professional was attended, or there was a mental health follow-up visit with an AHCP or primary care provider at the time after the baseline assessment. Mental health service use was also measured more stringently, excluding follow-up with the AHCP or primary care provider for a mental health concern, but no differences were found. The Actual Help-Seeking Questionnaire lists people who the adolescent may have sought help from for a personal or emotional problem and asks yes or no whether help was sought and to describe the problem it was sought for. The scale was modified to ask about the past 6 weeks. The original scale was also modified for clarity in a US health care setting as (3c) parent or guardian, (3e) mental health professional (counselor or social worker or psychologist or psychiatrist), (3f) helpline (phone number to call or text in crisis), and (3g) doctor or health care provider (doctor or provider you see for yearly physicals). In addition, 2 categories were added for coaches and religious persons (eg, priest, imam, and rabbi). At baseline, the General Help-Seeking Questionnaire [36] was used to ask about the intention to seek help from individuals on the aforementioned list, with a likelihood to seek help or advice for a personal or emotional problem from each individual on a 7-point Likert scale.

Other measures examined in adolescents and participating adults are shown in Table 1.

At baseline, both adolescents and parents were asked about their age, gender (male, female, transgender, and other with free text option), race, ethnicity, education (last grade level completed and any postgraduate education completed), and history of the adolescent ever receiving prior prescription medication or help from a mental health professional (eg, counselor or psychologist) for a personal or emotional problem [37]. If they answered yes to a prior visit with a mental health professional, they were asked what type of mental health professional, how many visits were completed, and how helpful the visits were on a Likert scale of 1 to 5. If prescribed a medication, they were asked about the helpfulness of the medication. Parents reporting a prior visit to a mental health professional were asked comparable questions. Socioeconomic status was assessed by asking yes or no questions about transportation (ease of finding transportation, own a car, or easy access to a car) and using the MacArthur socioeconomic ladder [38], comparing oneself with their community and the United States. Adolescents were asked about their sexuality with regards to identity, attraction, and behaviors. At baseline, there was an EHR review to assess what treatment was recommended at the patient visit. There were no changes to pilot trial assessments or measurements after the pilot trial commenced. There were no prespecified criteria used to judge whether to proceed with a future definitive trial.

#### Aim 4: Safety Protocols

We examined the appropriateness of the aforementioned implementation and effectiveness measures and kept the notation of any concerns for safety. The research team met weekly to ensure the data integrity and safety of the research participants. This included any concerns with regards to recruitment procedures, confidentiality, web data security, or safety. All identifiable and sensitive data were obtained through a secure database. All information stored on the intervention website was anonymous, except for the participants’ emails, which were stored on the back end (not visible to other participants). The participants were asked to agree to the ground rules as described earlier. Emergency contact information was obtained for all users even if they enrolled without their parents. The principal investigator, a physician with subspecialization in adolescent medicine, and the research team, consisting of RAs and graduate students with training in social work, underwent a 2-hour training session with regard to moderating the websites. Site moderation (reviewing any new users and new content, which only consisted of updates to a profile or comments posted to a blogpost) occurred at least every 3 hours during the day by reviewing a study email notifying the moderator on their mobile phone. If the participant were to make a reference to harming themselves, the moderator was to contact the participant or their emergency contact to gather more information, and in the event of the participant confirming suicidal thinking, history of an attempt, or plans to make an attempt in the future, the moderator was to contact the PI for further guidance as well as provide crisis resources and call emergency services as needed. This process is described in more detail in a separate publication [21].

### Sample Size

The sample size was determined based on the main pilot outcome, retention rate, which we determined would be at least 90%. This percentage was the goal to enhance trial efficiency with regard to budget and resources and to ensure that the study procedures were appropriate to achieve this retention rate. Our goal was to recruit sufficient adolescents to increase the likelihood of spontaneous peer-to-peer interactions occurring on the SOVA websites. For this goal, we desired a sample of 150 adolescents with 75 per arm and calculated a 95% CI for the retention rate to be between 85.2% and 94.8%. The study was terminated early after 38 adolescents were recruited because of meeting the pilot study goals, as described further in the *Discussion* section.

### Randomization and Blinding

Before study initiation, a statistician generated a permuted block randomization scheme and entered it into the REDCap database. Randomization was stratified by gender to account for anticipated low numbers of individuals specifying as cisgender male because of the clinic’s focus on providing contraception and reproductive health and to have even distribution between arms. The research team enrolled and consented participants, and if the criteria were met, the baseline survey was distributed. Once the baseline survey was completed by the adolescents, individuals were randomized by the research team using a REDCap module. AHCPs were blinded to the patients’ study arm. Separate research team members, who did not participate in enrollment and were limited from viewing enrollment or self-report survey data, conducted EHR extractions and were blinded to participant allocation to the study arm.

### Statistical Analysis Plan

Descriptive statistics (percentage for proportions and means and SDs for continuous measures) were used for all measures. We calculated the main pilot outcome, retention rate, and 95% CI. The baseline measures for covariate balance and outcome measures were summarized by the study arm. Each measure was examined by the study arm for changes from baseline to 6 weeks using 2 samples separately for adolescents and parents. Change scores versus mixed models were considered at 1 time point, 6 weeks, because that was the time point determined a priori to be of the highest clinical significance. A chi-square test was used to compare proportions for dichotomous outcomes. A *P* value of .05 was considered significant.

## Results

### Aim 1: Feasibility of Recruitment and Retention Strategies

**Figure 1:**
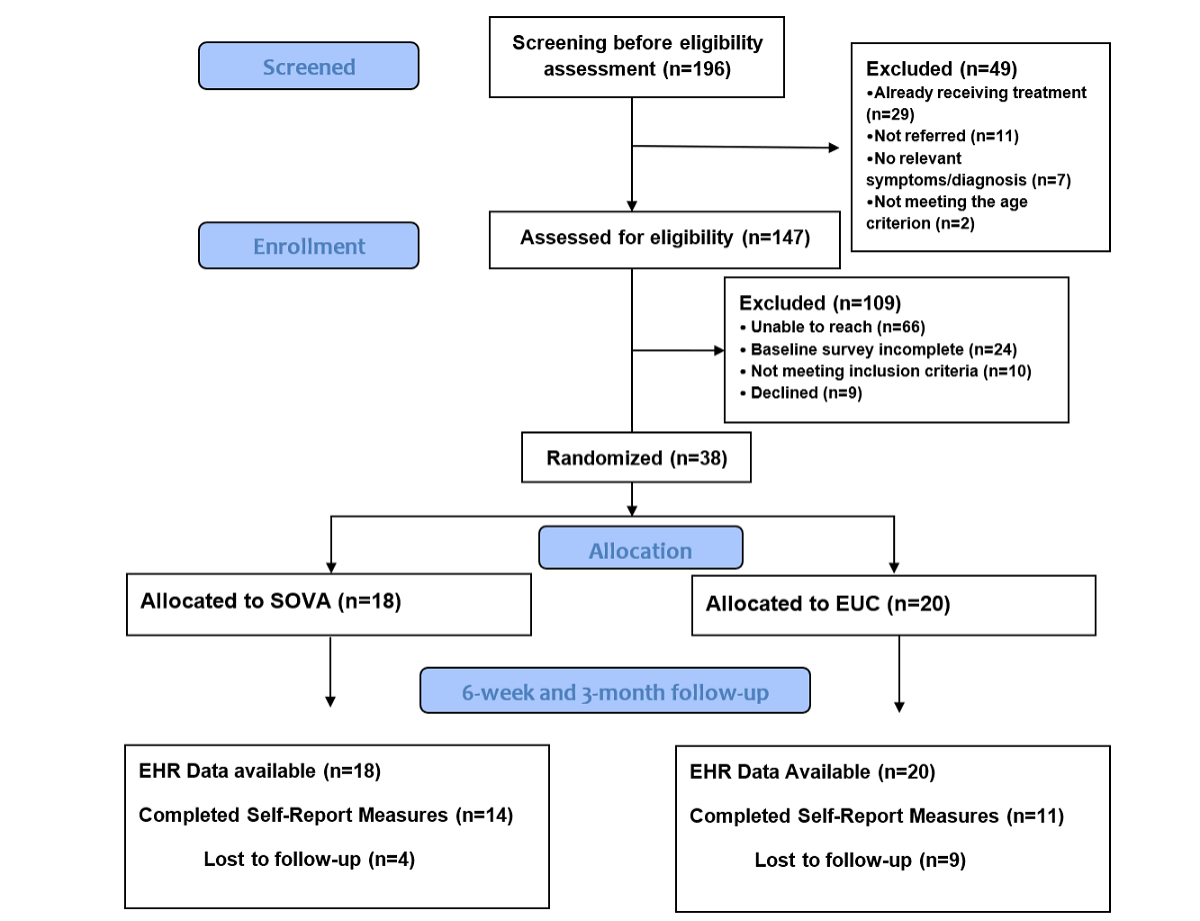
CONSORT (Consolidated Standards of Reporting Trials) diagram. EUC: enhanced usual care; SOVA: Supporting Our Valued Adolescents. EHR: electronic health record.

The CONSORT (Consolidated Standards of Reporting Trials) diagram of the study flow is shown in Figure 1 (Multimedia Appendix 2). A total of 196 adolescents were referred to the study by their health care providers. Of these 196 adolescents, 49 (25%) were excluded after the EHR chart review uncovered that they did not meet the study criteria. Of the 147 remaining participants, 66 (44.8%) could not be reached, 24 (16.3%) did not complete the baseline survey, 10 (6.8%) did not meet the inclusion criteria, and 9 (6.1%) declined study participation. The remaining 38 adolescents were randomized, of which 26 (68%) were recruited as a dyad with their parent and 12 (32%) were individually enrolled. Of the full sample (N=38), 18 (47%) were allocated to SOVA and 20 (53%) were allocated to EUC. Adolescents enrolling without a parent were equally distributed between SOVA (5/18, 28%) and EUC (7/20, 35%) arms. Of the 38 participants, 25 (66%, 95% CI 51%-81%) completed the 6-week measures. There were few differences between those at baseline who completed and those who did not complete the 6-week measures. Study participants who did not complete the 6-week measures were more likely to, at baseline, say they had a depressed mood on most days in the last year (12/13, 92%) compared with those who completed the 6-week measures (15/25, 60%; *P*=.04), but there were no differences between total PHQ-9 scores; have lower anxiety literacy (mean 7.8, SD 3.5 vs mean 9.7, SD 2.4, *P*=.05); and report that asking for but not getting help had occurred to them in the past few weeks (3/13, 23% vs 0/25, 0%; *P*=.01). There were 65 adolescents who consented to but did not enter the study either because they did not complete the baseline or they were recruited earlier in the study when parental enrollment was required, but their parents could not be reached. There were no statistically significant differences between the group that consented but did not enter the study and the group that entered the study (N=38) in terms of patient age, depressive symptom score, or anxiety symptom score. They were less likely to have a parent who desired to enroll in the study (21/65, 36%) compared with those entering the study (26/38, 68%; *P*=.002), although some adolescents (before the first 17 were enrolled) were not offered entry into the study because of earlier study entry requirements requiring parental enrollment. When comparing based on condition, 55% (11/20) of the EUC arm sample completed the 6-week measures as compared with 78% (14/18) of the SOVA arm, *P*=.14, *χ*^2^_1_=2.2.

### Baseline Characteristics

The baseline characteristics of the sample are available in Table 2 briefly and in detail in Tables S1 and S2 in Multimedia Appendix 3. On average, adolescents were approximately aged 16 years, and their parents were approximately aged 44 years in the SOVA arm and 47 years in the EUC arm. Most adolescents in the EUC arm were identified as female (17/20, 85%), while in SOVA, there were also 33% (6/20) who identified as transgender or other gender. Approximately half of each sample (60% in EUC and 50% in SOVA) identified as 100% heterosexual. The adolescent sample was about three-fourths White, and none were Hispanic or Latino. A substantial number of adolescents had prior history of receiving psychotherapy (70% in EUC and 50% in SOVA), and few had received medication before (30% in EUC and 17% in SOVA). The parents were all female and mostly White.

**Table 2:** Baseline characteristics for the adolescent and parent study samples.

Baseline characteristic		Adolescent			Parent	
		EUC^a^ (n=20)	SOVA^b^ (n=18)	EUC (n=13)		SOVA (n=13)
Age (years), mean (SEM^c^)		15.9 (1.7)	16.1 (1.6)	47.2 (5.2)		43.8 (6.1)
**Gender identity,** **n (%)**						
	Male	2 (10)	0 (0)	0 (0)		0 (0)
	Female	17 (85)	12 (67)	13 (100)		13 (100)
	Transgender	0 (0)	2 (11)	0 (0)		0 (0)
	Other or >1	1 (5)	4 (22)	0 (0)		0 (0)
**Sexual identity,** **n (%)**						
	Heterosexual (straight)	12 (60)	9 (50)	N/A^d^		N/A
	Mostly heterosexual	4 (20)	2 (11)	N/A		N/A
	Bisexual	2 (10)	4 (22)	N/A		N/A
	Mostly homosexual	1 (5)	2 (11)	N/A		N/A
	Homosexual (gay)	1 (5)	1 (6)	N/A		N/A
**Race,** **n (%)**						
	White	15 (75)	13 (72)	11 (85)		13 (100)
	Black or African American	2 (10)	1 (6)	2 (15)		0 (0)
	>1 race	1 (5)	4 (22)	0 (0)		0 (0)
	Asian	1 (5)	0 (0)	0 (0)		0 (0)
	American Indian or Alaska native	1 (5)	0 (0)	0 (0)		0 (0)
**Ethnicity,** **n (%)**						
	Hispanic or Latino	0 (0)	0 (0)	1 (8)		0 (0)
**Symptoms, mean (SEM)**						
	Depressive symptoms (0-27)	11.8 (5.5)	10.8 (3.5)	N/A		N/A
	Anxiety symptoms (0-21)	11.0 (5.8)	8.9 (4.2)	N/A		N/A

^a^EUC: enhanced usual care.

^b^SOVA: Supporting Our Valued Adolescents.

^c^SEM: SE of the mean.

^d^N/A: not applicable.

### Aim 2: Implementation Outcomes and Intervention Fidelity

We found limited self-reported engagement in the treatment arm, with only 43% (6/14) of the adolescents who completed the 6-week measures and were randomized to SOVA reporting they ever accessed the intervention. While 14% (2/14) thought they were in the EUC arm, the 6 who knew they were in the SOVA arm cited free text reasons for not looking at the site, which were mostly due to forgetting (2/14, 14%) and not having time (2/14, 14%; 1 individual cited both forgetting and not having time), 7% (1/14) were not sure and 14% (2/14) said they did not know how or “it’s confusing” but did not specify whether the study or the intervention was confusing. There were few differences at baseline between adolescents who visited the site and those who did not. A few notable differences were that those visiting the site in the first 6 weeks experienced higher perceived stigma (mean 27, SD 6.1 vs mean 15.7, SD 6.1; *P*=.01) and fewer negative feelings toward antidepressants (mean 6.4, SD 5.1 vs mean 13.3, SD 5.1; *P*=.05). At 6 weeks, we asked all adolescents if they were in the arm with the website (without providing the link or name), and if they answered yes, they were asked how often they accessed it; 2 (18%) adolescents in the EUC arm reported they had accessed the site at that time. At 12 weeks, study end, we asked all the users if they had accessed the sites. In the EUC arm, 27% (3/11) of adolescents and in the SOVA arm, 45% (6/14) of adolescents reported accessing the site, showing evidence of some crossover, although out of the 3 accessing SOVA in the EUC arm, only 1 accessed the site more than once. Of the adolescents reporting accessing the site, 22% (2/9) self-reported accessing it once, 56% (5/9) accessed it 2 to 5 times, and 22% (2/9) accessed it 5 to 10 times. Among parents, at 6 weeks, 60% (6/10) reported having accessed the site in the SOVA arm and 20% (2/10) in the EUC arm reported accessing it. At 12 weeks, 30% (3/10) of the parents in the SOVA arm and 11% (1/9) of the parents in the EUC arm said they had accessed the parent sites. Parents reported reasons for not accessing the site because of forgetting and prioritizing other aspects of their lives, as evident in these example free text responses: “I have been curious to visit the website but have not made it a priority and I probably should” and “I have a son with autism and just don’t have time.” Of note, no one used the mobile app option.

During the study timeframe (March 7, 2018, to June 5, 2020), 461 unique adolescents were provided with the “Stress and Worry” patient education handout. The proportion of those who were initially interested in the study and were screened was 42.2% (195/461). Out of 11 AHCPs answering the poststudy survey, 7 (63%) reported including the “Stress and Worry” patient education for end-of-visit depart summaries. Of the 37% (4/11) who did not use it, they cited forgetting its existence and a workflow concern with switching to a new EHR three-fourths of the way through the trial. Of the 6 respondents answering follow-up questions about the patient education material, on the 0 to 3 scale, most found it clinically useful (mean 2.8, SD 0.4), intuitively appealing (mean 2.8, SD 0.4), and made sense (mean 2.7, SD 0.8). When asked if they thought colleagues were happy using the “Stress and Worry” patient education, 67% (4/6) of participants noted to a very great extent, 17% (1/6) to a moderate extent, and 17% (1/6) not at all (mean 2.33, SD 0.49). The majority reported that using education was not something they did only because they thought it was required (mean 0.5, SD 0.8). Most noted that patients did not bring the study up to them (mean 0.7, SD 0.8), but social workers did to a moderate extent (mean 2.0, SD 1.0). Suggestions for improvement included setting reminders to use the patient education or automating its inclusion in yearly visit depart summaries, increasing advertising, and periodically providing updates about the form, as well as having information in the EHR denoting which patients were in the study.

### Exploratory Aim 3: Between-Arm Comparisons of Clinical Outcomes

At 6 weeks, 61% (11/18) of adolescents randomized to SOVA received mental health treatment compared with 50% (10/20) of adolescents randomized to EUC (*P*=.53), and at 12 weeks, 83% (15/18) of adolescents randomized to SOVA received mental health treatment compared with 50% (10/20) of adolescents randomized to EUC (*P*=.03), where receipt of treatment was measured by a combination of either adolescent or parent self-report and a blinded manual EHR extraction. Blinded manual EHR extraction included individuals who did not complete the 6-week measures, making it possible to examine the full sample despite their survey nonresponse.

It is important to note that these were exploratory comparisons. For the most part, we found no difference in the between-arm comparisons of 6-week outcomes in an intention-to-treat analysis between those randomized to the SOVA intervention and those randomized to the EUC arm (Table 3). Some noted differences included a decrease in total stigma, increase in social support, and decrease in anxiety in the EUC group as compared with the SOVA group for adolescents, and a decrease in stigma as compared with baseline for the EUC group as compared with the SOVA group for parents. In both SOVA and EUC, 1 adolescent who at baseline did not perceive a need for services, at 6 weeks said they did need services, and there was no statistically significant difference for changes in perceived need (0.47), although on the General-Practice Users Perceived-Need Inventory scale, adolescents in EUC were more likely (8/11, 73%) compared with those in SOVA (3/14, 57%; *P*=.02) to not want help because of a preference for self-management.

As there was significant crossover between arms, as well as lack of engagement within the treatment arm, we conducted a per-protocol analysis for adolescents to see if any between-arm differences would remain. We compared those who self-reported that they accessed SOVA (8/25, 32%) at least once (including 2 in the EUC arm) with those who did not (17/25, 68%). We found no clinically or statistically significant differences, except an increase in peer functioning in the SOVA group versus EUC (mean 2.0, SD 1.3 vs mean −0.2, SD 2.3; *P*=.02; Table 4).

**Table 3:** Six-week exploratory comparison in change scores between SOVA^a^ and EUC^b^.

Change in scores			Adolescents							Parents				
			EUC (n=11), mean (SD)		SOVA (n=14), mean (SD)		*P* value		EUC (n=11), mean (SD)			SOVA (n=10), mean (SD)		*P* value
**Stigma**			−5.6 (8.1)		0.4 (5.7)		.04		−2.3 (5.8)			4.1 (6.8)		.03
	Personal stigma	−2.3 (4.3)		0.2 (2.9)		.10		1.2 (3.9)			2.7 (2.9)		.33	
	Perceived stigma	−3.4 (5.8)		0.2 (3.8)		.08		−3.4 (4.6)			1.4 (7.0)		.07	
Acceptance of antidepressant use			−0.6 (4.0)		−0.6 (2.8)		1		0.8 (2.8)			0.6 (4.7)		.90
Worry about antidepressant			−1.6 (4.8)		0.1 (6.0)		.45		.45 (3.5)			0.8 (8.8)		.91
Barriers to adolescent seeking help from a therapist			1.3 (19.9)		−6.3 (20.5)		.36		−22.2 (10.1)			−10.2 (18.7)		.08
**Knowledge**														
	Depression knowledge	0 (4.4)		−1.4 (2.8)		.35		−6.0 (2.4)			−4.9 (2.7)		.34	
	Anxiety knowledge	0.6 (4.2)		0.4 (2.4)		.88		1.6 (2.0)			0.6 (3.0)		.36	
Social support			13.1 (14.2)		−10.5 (29.2)		.02		3.1 (12.7)			−2.5 (10.9)		.30
**Parent-adolescent communication quality (20-100)**			0.9 (7.0)		−0.7 (4.8)		.50		1.9 (6.0)			0.3 (9.6)		.65
	Openness of communication	1.7 (6.5)		−1.0 (6.2)		.30		−0.2 (6.5)			−0.3 (4.6)		.96	
	Extent of communication	−0.8 (4.5)		0.3 (7.4)		.67		2.1 (7.3)			0.6 (5.8)		.61	
**Symptoms**														
	Depressive symptoms	−3.2 (3.7)		0.8 (6.8)		.09		N/A^c^			N/A		N/A	
	Anxiety symptoms	−4.0 (5.6)		−0.1 (3.6)		.04		N/A			N/A		N/A	
**Adolescent functioning**														
	(Parent: Columbia Scale)	N/A		N/A		N/A		−1.4 (6.2)			0.9 (3.8)		.32	
	General functioning	0.0 (5.8)		−0.1 (5.0)		.95		N/A			N/A		N/A	
	Family functioning	−0.3 (2.7)		−0.4 (3.3)		.95		N/A			N/A		N/A	
	Peer functioning	0.3 (1.9)		0.6 (2.6)		.70		N/A			N/A		N/A	
Parent-adolescent relationship quality			15.3 (4.6)		13.7 (4.2)		.38		16.6 (4.3)			14.4 (4.7)		.28

^a^SOVA: Supporting Our Valued Adolescents.

^b^EUC: enhanced usual care.

^c^N/A: not applicable.

**Table 4:** Six-week per-protocol analysis, comparing change scores between adolescents accessing the SOVA^a^ intervention and those who did not access it (N=25).

Change in scores		Did not access SOVA (n=17), mean (SD)	Accessed SOVA (n=8), mean (SD)	*P* value
**Stigma (total)**		−2.6 (8.1)	−1.5 (6.1)	.74
	Personal stigma	−0.8 (4.4)	−1.1 (2.0)	.83
	Perceived stigma	−1.8 (5.1)	−0.4 (4.9)	.51
Acceptance of antidepressant use		−1.1 (3.6)	0.4 (2.3)	.30
Worry about antidepressant		−1.9 (5.2)	2.0 (5.2)	.09
Barriers to adolescent seeking help from a therapist		−1.7 (18.6)	−5.7 (24.4)	.65
**Knowledge**				
	Depression knowledge	−0.6 (4.0)	−1.1 (2.7)	.73
	Anxiety knowledge	0.7 (3.7)	0.1 (2.2)	.69
Social support		−0.2 (28.4)	0.0 (22.7)	.99
**Parent-adolescent communication quality**		1.4 (5.8)	−2.9 (5.0)	.09
	Openness of communication	0.8 (6.7)	−1.1 (5.9)	.49
	Extent of communication	0.5 (6.0)	−1.8 (6.8)	.40
**Symptoms**				
	Depressive symptoms	−0.7 (6.8)	−1.6 (3.3)	.71
	Anxiety symptoms	−2.4 (5.5)	−0.6 (3.2)	.42
**Adolescent functioning**				
	General functioning	−0.8 (5.4)	1.5 (4.8)	.31
	Family functioning	−0.6 (3.3)	0.2 (2.4)	.53
	Peer functioning	−0.2 (2.3)	2.0 (1.3)	.02
Parent-adolescent relationship quality		0.3 (3.1)	−0.9 (3.0)	.39

^a^SOVA: Supporting Our Valued Adolescents.

### Aim 4: Safety and Follow-up

The safety protocol was found to be adequate as there were no events indicative of safety concerns or breaches of data integrity or confidentiality. No posts were removed because of safety issues. No harm or unintended effects were noted in either group. Recruitment began in March 2018 and the last follow-up time point for the last participant was April 2020. The pilot trial was ended because of expiration in funding, ceasing in-person recruitment because of the start of the COVID-19 pandemic and the Pennsylvania Governor’s stay-at-home order, as well as because of obtaining enough data to determine the feasibility of recruitment and retention findings.

## Discussion

### Principal Findings

The main findings of this pilot trial highlight the difficulties in conducting research with adolescents for technology-based mental health studies in the primary care setting. We met 25% of our sample size goal. The goal of 150 adolescents was based on the desire to observe spontaneous peer-peer interaction on the web and was not necessary to meet our pilot outcomes, which were to examine the feasibility of and refine recruitment and retention strategies; therefore, the study was terminated before reaching that goal. This termination was also contributed to by the prohibition of in-person recruitment during the start of the COVID-19 pandemic and budgetary constraints. We found that implementing patient education by introducing the study into the provider workflow was feasible and acceptable. This likely contributed to the ease of referral to the study, as we observed a large number of referrals (almost 500). Had everyone who had consented completed the baseline survey, we would have met 85% of our sample size goal. This led us to understand that the main challenge of this pilot study was retention to study initiation after completing consent. To increase the reach of this mental health intervention to youth, especially those whose parents may be unaware of their need for mental health referral—as parental perceived need is one of the intervention targets but may limit enrollment—we amended the study design to not require parental enrollment or permission. We found that this was detrimental to initial retention because participants enrolling without a parent were more difficult to reach and less likely to complete the baseline survey. We found that only approximately half of the participants in the intervention arm reported ever viewing the sites. Similarly, less than half of the parents reported accessing the site. Our parent stakeholder group expressed that some parents desire to use the site only when their child is symptomatic as a resource when they need information but not on a routine basis.

We found a signal for greater mental health service use at 12 weeks but not at 6 weeks. At 6 weeks, half of those randomized to EUC had accessed mental health treatment as compared with approximately 60% of those randomized to SOVA, but at 12 weeks, EUC remained at half, while 83% of those randomized to SOVA accessed treatment. Despite an approximately two-thirds survey response rate, we were able to supplement our data for mental health service use from the EHR to examine the full sample. Much of the sample had had prior mental health treatment, and so their perceived need for further treatment may have been tempered by whether they felt prior treatment was helpful. This factor may be important to measure in a future trial. This finding of greater uptake of mental health treatment is tempered as the study was not powered to examine this main outcome, the arms were not balanced especially with regard to gender minorities, and the study was limited by less than expected intervention engagement as well as some crossover. Finding this difference does suggest that SOVA warrants further study to understand its potential benefit on mental health service use in adequately powered samples. Examining outcomes at 12 weeks may be more meaningful than at 6 weeks, as even if mental health services are desired, multiple nonattitudinal barriers may influence difficulty with timely use of services, such as insurance, timing, appointment availability, and scheduling barriers. An intervention such as SOVA, which continues to be available during these difficulties, may help maintain the motivation to and perceived need to seek treatment. Although in this trial, we examined the uptake of treatment in individuals whose medical provider recommended they initiate treatment and had not been in treatment for the previous 3 months, a secondary outcome of interest in future trials would be reinitiation of treatment and continued adherence to treatment.

In this pilot sample, we did not find statistically significant differences in changes in target outcomes, which were only examined at 6 weeks, for the most part, and for those we did find (ie, stigma, social support, and anxiety), they appeared to go in the opposite of the proposed hypothesized direction. It is important to note that examining differences in these clinical outcomes was preliminary and very exploratory. The differential effects in the 2 groups were likely random because of the small sample size in this pilot study and less likely because of the intervention, as when a per-protocol analysis was performed, those differences disappeared. Some differences that were seen may have had more to do with the likelihood of finding a difference based on the number of tests done and that randomization was not successful because of the small sample size. In addition, these differences disappeared in a per-protocol analysis comparing adolescents who accessed SOVA with those who did not at 6 weeks. In this analysis, there was only a small increase in peer functioning in SOVA as compared with that in EUC. Because of the small sample size and exploratory nature of the study, we did not have the power to detect differences. A lack of finding these differences may be due to a lack of intervention engagement, lack of power to detect them, or intervention crossover or that the intervention mechanism is not explained by these targets.

For this study, we deemed the training procedures for safety protocols to be adequate and feasible as we were able to train multiple social work graduate students to take on this role and split its demands, including managing the website, totaling about the equivalent of 1 part-time employee per week. We did not have any safety concerns. As participants were all patients of the same clinic, providers were available to contact if, at screening, participants were noted to have high scores for depression or anxiety symptoms. There were low rates of data missingness for those who responded, the main concern being study initiation and retention after consent.

### Strengths and Limitations

There are a few notable strengths of this study. First, despite its pilot nature, because we had already done a preimplementation study [39], we were able to gather further data on parts of an implementation strategy during this trial. We were not able to test the full strategy, as it would have risked crossover for participants not randomized to the intervention. Another strength is the pragmatic nature of this trial with regard to testing a technology intervention in a real-world setting, relying on clinicians and social workers to introduce the study, and maintaining adolescent autonomy in decision-making about their mental health by allowing them to autonomously enter the study with a waiver of parental permission. The same strengths of the study design contributed to consequences that resulted in limitations. Owing to its testing in a real-world setting and the main goal being treatment uptake, the timing of introducing the intervention immediately after referral to treatment is important. If delayed, understanding whether the intervention directly contributes to treatment initiation is difficult. When factoring in the busyness of clinic flow, recruitment can be difficult. Despite this, we were able to receive almost 500 referrals, and the main limitation of this study was a reduced sample size because of the failure of those who consented to the study to initiate the baseline survey and subsequently be randomized. We found that 1 factor influencing this was the waiver of parental permission. Although waiving parental permission facilitated reaching the target population—adolescents who may not initiate mental health treatment because of lack of parental involvement, engagement, or awareness of symptoms—it limited the final sample size. Although we stratified arms based on gender to ensure that an equal number of the anticipated low number of cisgender males would be equally distributed, we had unbalanced arms with regard to gender minorities, with 6 individuals who were transgender or other>1 gender in the SOVA arm and 1 individual who was other or >1 gender in the usual care arm. During the time of the study, the clinic began a new program for gender and sexual development, and the number of gender minority individuals increased; therefore, this was not planned for in the original study design. An exploratory logistic regression with 3-month mental health treatment as the outcome when controlling for gender found a small decrease in the statistical significance found in the full sample by 0.02. This informs to stratify by all genders in future studies to ensure equal balance across arms.

Furthermore, about half of the sample recruited to the SOVA intervention reported not viewing the intervention. There was some crossover reported between arms, but this was minimal, as only 1 participant in the control arm accessed the site more than once. We were limited in the technology used for this study to measure user engagement as individuals could view site content without logging in or blurbs about articles in an email newsletter, limiting our ability to capture their use if they never logged in. We instead relied on self-report for both reports of site use and crossover, and hence, we cannot be sure of the accuracy of site use and, therefore, intervention engagement. Throughout most of the study, participants received emails notifying them of new website content, as SMS text messaging research participants was not yet a standard communication because of privacy concerns. Toward the end of the study, as texting participants became more of a research norm, we began a protocol for increasing intervention engagement by tracking user log-ins and sending SMS text message suggestions to view articles to participants who did not log in. Future iterations will further personalize article recommendations to participants based on baseline survey data (eg, someone who lists cost as a barrier will be sent an article about the cost of mental health care). Future studies should seek opportunities for increased funding to enable the use of more sophisticated user metrics. These metrics also become more accessible to researchers with advancements in technology. However, in our initial usability study, user engagement was higher than the typical rates in similar studies [20]. We believe that the individual randomization design and not introducing participants to the intervention from the start may have also contributed to low engagement. One factor to consider in efforts to increase intervention engagement is that, although not significant, we did find that less of the EUC group (55%) completed the 6-week measures as compared with the SOVA (78%) group, suggesting that having an attention control that also has some ongoing engagement may decrease retention differences between arms.

Another limitation is we did not adjust for multiple comparisons. As this was a pilot trial, analyses of comparisons were solely exploratory and, although adjusting for multiple comparisons would avoid type I error, we were more interested in not inflating type II error for the purposes of informing which measures may be the most important to examine in a future fully powered trial. In addition, currently, the SOVA intervention is only in English, and therefore, we had to exclude any non–English-speaking participants. This did not lead to any exclusion in this study, and if we find effectiveness in a fully powered trial, we will pursue language and cultural adaptations of the intervention.

### Implications for Future Research

Although referrals to this pilot study were adequate, retention and engagement were lacking. For adolescents with mental health concerns, some of the same barriers to initiating mental health care, such as lack of motivation or lack of parental support, may also act as barriers to enrolling in research. For example, adolescents considering participation in HIV research may choose not to enroll because of stigma and requirements for parental consent [40]. For some sensitive issues, adolescent participation in research may not be possible without waiving parental permission [41]. During the trial, the recruitment rate actually increased after passing a waiver for parental permission and not requiring adolescents to enter the study as a dyad. This change is conceptually consistent, as adolescents who have poor communication with their parents are a SOVA target population but may be more difficult to recruit if parental permission is required. In addition, including a parental waiver more closely simulates real-world scenarios, as in almost half of the states of the United States [42] and about one-third of countries responding to a World Health Organization child policy survey [43], adolescents may provide consent for their own mental health care. We found during our study that, in fact, the research procedures were of minimal risk, which may help influence other ethics boards to approve similar waivers. On the contrary, a lack of parental enrollment may have negatively influenced retention and engagement.

The limitations of retention and engagement in this pilot trial are not necessarily a reflection of the intervention itself being difficult to engage with. Recently, we found that adolescents and young adults have been very interested in contributing content to SOVA sites [44,45], especially during the COVID-19 pandemic, during which social isolation increased in adolescents.

A different study design that does not require individual randomization would improve retention and engagement, as we would have the freedom to expose individuals to the intervention from the point of study recruitment, instead of requiring them to complete an initial lengthy survey before learning about whether they would be randomized to the intervention. Although a cluster trial design is more complex and resource intense, it would allow for clustering based on the primary care clinic as a unit. This clinic-based recruitment could have the added benefit of onboarding an entire primary care clinic with additional implementation strategies, as recommended by primary care providers participating in our preimplementation study [39]. In particular, this would include distributing materials about the intervention to all adolescents presenting to the practice, regardless of whether they enroll in the study. Viewing the intervention before enrollment may increase interest in the study and enhance engagement. Our target population included adolescents and parents who may be resistant to speaking to their primary care provider about mental health symptoms and answer a screen falsely to avoid such a conversation. Because of the study inclusion criteria necessitating referral to therapy, these adolescents may never be exposed to the intervention if there is individual randomization. Instead, in a cluster design, all adolescents and parents visiting the practice site would be provided information about SOVA. Recruitment and study initiation rates may be enhanced if all adolescents and parents know they will receive the intervention. We hypothesize that prior pilot trial recruitment would have been more successful if all knew they would receive the intervention, as during the recruitment and consent process, it was evident that they perceived the site as a potential benefit and were excited about using it but were disappointed when they knew they may not initially receive it. However, this design may limit evaluating immediate before and after changes in potential target mechanisms (ie, stigma and other barriers) if participant data are difficult to capture before the initial viewing of the site. However, we may still be able to capture a dose effect, and, importantly, we would be able to understand whether practices in the intervention arm have improved the uptake of mental health treatment in adolescents with depression or anxiety as compared with treatment as usual.

### Conclusions

In conclusion, in this pilot trial of a peer support website intervention for adolescents with depression or anxiety, we found lower-than-expected study enrollment after recruitment. Although study retention may be limited by not requiring parental enrollment and parental permission, the tradeoff of enrolling adolescents who may have more difficulty seeking mental health services because of a lack of parental support may enhance generalizability and reach to the target population. Therefore, future trials will continue to waive parental permission but account for expected attrition from consent to randomization. We will also plan to use more engaging methods to reach adolescents, such as SMS text messaging as opposed to email, and incorporate automatic notifications. We will use a cluster trial design to increase the initial interest in the study intervention, as this may enhance intervention engagement from the start as compared with individual randomization. In addition, we found a signal for greater uptake of mental health treatment in adolescents using SOVA, a peer support website intervention for adolescents with depression or anxiety and their parents. We determined that our safety protocols were adequate and that no adverse events occurred. This pilot study informs a larger trial in planning for attrition, pairing-down salient measures, waiving parental permission, informing a protocol for increasing website engagement, and refining parameters for an automated EHR extraction.

## Appendix

Multimedia Appendix 1 “Stress and Worry” Patient Handout.

Multimedia Appendix 2 CONSORT-eHEALTH checklist (V 1.6.1).

Multimedia Appendix 3 Supplemental tables.

## Abbreviations

AHCP: adolescent health care provider
CONSORT: Consolidated Standards of Reporting Trials
EHR: electronic health record
EUC: enhanced usual care
GAD-7: Generalized Anxiety Disorder Questionnaire-7
PHQ-9: Patient Health Questionnaire-9
RA: research assistant
REDCap: Research Electronic Data Capture
SOVA: Supporting Our Valued Adolescents
