# Supplementary material for: A Social Media Website (Supporting Our Valued Adolescents) to Support Treatment Uptake for Adolescents With Depression or Anxiety: Pilot Randomized Controlled Trial
Source: JMIR Ment Health. 2022 Oct 7;9(10):e35313. doi: 10.2196/35313 (PMC9587493; doi:10.2196/35313)
Supplement: Multimedia Appendix 1 [file mental_v9i10e35313_app1.docx]

**STRESS AND WORRY**

Today we spent some of your visit talking about STRESS and WORRY. Everyone can experience these but I think these are starting to affect your life in a way that could be harmful to your health, so I want to help you take steps to get better control over stress and worry.

Here are some specific recommendations for you (in parentheses find some websites where you can get more information about these issues):

___ Working on some more self-care - specifically:

___ Sleep ([sleepeducation.org/essentials-in-sleep/healthy-sleep-habits](http://www.sleepeducation.org/essentials-in-sleep/healthy-sleep-habits))

___ Nutrition (choosemyplate.gov/)

___ Exercise (safeteens.org/nutrition-exercise/exercise-fitness/)

___ Technology (healthychildren.org/English/family-life/Media/Pages/Points-to-Make-With-Your-___ Teen-About-Media.aspx)

___ Your health (youngwomenshealth.org or youngmenshealthsite.org)

___ Starting a new medication (<http://parentsmedguide.org/>)

___ Seeing a professional to talk to (<https://www.psychologytoday.com/>)

___ Coming back to see me again to check in

___ Other:

If YOU want to learn more about depression and anxiety, please check out these websites:

Depression: [nimh.nih.gov/health/publications/teen-depression/index.shtml](https://www.nimh.nih.gov/health/publications/teen-depression/index.shtml)

Anxiety: [nimh.nih.gov/health/publications/generalized-anxiety-disorder-gad/index.shtml](https://www.nimh.nih.gov/health/publications/generalized-anxiety-disorder-gad/index.shtml)

If YOUR PARENT wants to learn more about depression and anxiety in adolescents, they can go to:

Depression: [aacap.org/AACAP/Families_and_Youth/Resource_Centers/Depression_Resource_Center/Home.aspx](https://www.aacap.org/AACAP/Families_and_Youth/Resource_Centers/Depression_Resource_Center/Home.aspx)

Anxiety: [aacap.org/AACAP/Families_and_Youth/Resource_Centers/Anxiety_Disorder_Resource_Center/Home.aspx](https://www.aacap.org/AACAP/Families_and_Youth/Resource_Centers/Anxiety_Disorder_Resource_Center/Home.aspx)

IF YOU ARE EXPERIENCING CRISIS **(IF NOT, PUT THIS INFO IN YOUR PHONE JUST IN CASE!!!):**

If you feel like you may be at risk for hurting yourself or someone you love, please let someone know. If you have a supportive adult you can trust, let them know. Most counties in the U.S. have a phone number you can call if you are in crisis and the person on the other line will help you figure out what to do.

In Pittsburgh, Allegheny County, that number is 1.888.796.8226

If you live somewhere else, you can call the National Suicide Prevention Lifeline at 1.800.273.8255 and they will connect you with your local crisis center.

Otherwise you can call 911.

Other resources include:

Crisischat.org is an online crisis resource from the National Suicide Prevention Lifeline.

Two other crisis sites include: imalive.org and crisistextline which can also be contacted by texting “START” to 741-741

A STUDY YOU MIGHT BE INTERESTED IN:

**You may be interested in a research study that may help you learn some more about stress and worry and help us remind you what we talked about today.**

Make sure to fill out the bookmark you got in clinic and give it to anyone in clinic OR email socialmediastudy@chp.edu with the title "Want Info on Stress and Worry Study" with your name, your phone number, your parent or guardian's name, and their phone number and the study team will get back to you! You and your parent would both get compensated for being in the study. There is a 20-30 minute survey in the beginning and one in 6 weeks, and then a short survey 3 months from now.

Thanks for thinking about it! Your participation might help someone like you in years to come! So that person thanks you too!!
