# Supplementary material for: A Social Media Website (Supporting Our Valued Adolescents) to Support Treatment Uptake for Adolescents With Depression or Anxiety: Pilot Randomized Controlled Trial
Source: JMIR Ment Health. 2022 Oct 7;9(10):e35313. doi: 10.2196/35313 (PMC9587493; doi:10.2196/35313)
Supplement: Multimedia Appendix 3 [file mental_v9i10e35313_app3.docx]

**Supplemental Table 1 Full Baseline Characteristics for the Adolescent Study Sample**

| Baseline Characteristic | EUC | | SOVA | |
| --- | --- | --- | --- | --- |
|  | (n=20) | | (n=18) | |
|  | Mean (SEM) | N (%) | Mean (SEM) | N (%) |
| **Age** | 15.9 (1.7) |  | 16.1 (1.6) |  |
| **Gender Identity** |  |  |  |  |
| Male |  | 2 (10%) |  | 0 (0%) |
| Female |  | 17 (85%) |  | 12 (67%) |
| Transgender |  | 0 (0%) |  | 2 (11%) |
| Other or more than one |  | 1 (5%) |  | 4 (22%) |
| **Sexual Identity** |  |  |  |  |
| 100% heterosexual (straight) |  | 12 (60%) |  | 9 (50%) |
| Mostly heterosexual |  | 4 (20%) |  | 2 (11%) |
| Bisexual |  | 2 (10%) |  | 4 (22%) |
| Mostly homosexual |  | 1 (5%) |  | 2 (11%) |
| 100% homosexual (gay) |  | 1 (5%) |  | 1 (6%) |
| **Sexual Attraction** |  |  |  |  |
| Not at all sexually attracted to the same sex |  | 12 (60%) |  | 9 (50%) |
| Slightly attracted to the same sex |  | 6 (30%) |  | 5 (28%) |
| Extremely attracted to the  same sex |  | 2 (10%) |  | 4 (22%) |
| **Race** |  |  |  |  |
| White |  | 15 (75%) |  | 13 (72%) |
| Black or African American |  | 2 (10%) |  | 1 (6%) |
| More than one race |  | 1 (5%) |  | 4 (22%) |
| Asian |  | 1 (5%) |  | 0 (0%) |
| American Indian or Alaska Native |  | 1 (5%) |  | 0 (0%) |
| **Ethnicity** |  |  |  |  |
| Hispanic or Latino |  | 0 (0%) |  | 0 (0%) |
| **Education (grade level)** |  |  |  |  |
| 7-8^th^ |  | 5 (25%) |  | 7 (39%) |
| 9-10^th^ |  | 7 (35%) |  | 3 (17%) |
| 11-12^th^ |  | 5 (25%) |  | 5 (28%) |
| Graduated High School |  | 3 (15%) |  | 3 (17%) |
| **Health Insurance Coverage^a^** |  |  |  |  |
| Private |  | 7 (54%) |  | 4 (33%) |
| Medicaid/CHIP |  | 5 (38%) |  | 4 (33%) |
| None |  | 0 (0%) |  | 1 (8%) |
| **Access to Transportation** |  | 18 (90%) |  | 14 (78%) |
| **Social Status Ladder – Community (1-10)** | 5.7 (1.8) |  | 4.8 (2.1) |  |
| **Social Status Ladder – United States (1-10)** | 5.7 (2.1) |  | 4.2 (2.1) |  |
| **Prior History of Receiving Psychotherapy** |  | 14 (70%) |  | 9 (50%) |
| Number of Visits with Professional if any | 22.1 (32.4) |  | 10.7 (17.8) |  |
| How helpful prior visits were (1-5) | 2.3 (0.9) |  | 1.5 (0.5) |  |
| **Prior History of Receiving Medication** |  | 6 (30%) |  | 3 (17%) |
| How helpful medication was (1-5) | 2.4 (0.6) |  | 1.7 (0.6) |  |
| **Stigma – total (0-72)** | 25.9 (8.1) |  | 25.3 (10.6) |  |
| Personal Stigma (0-36) | 6.9 (4.9) |  | 6.9 (5.0) |  |
| Perceived Stigma (0-36) | 19.0 (8.6) |  | 18.3 (8.3) |  |
| **Acceptance of Antidepressant Use (RAUQ) (0-18)** | 13.0 (4.6) |  | 13.9 (3.6) |  |
| **Worry about Antidepressant (AMS) (0-24)** | 12.0 (5.6) |  | 10.3 (5.1) |  |
| **Barriers to Adolescent Seeking Help from a Therapist (11-66)** | 28.6 (8.2) |  | 31.8 (9.4) |  |
| **Depression Knowledge (0-22)** | 12.4 (3.7) |  | 12.5 (2.8) |  |
| **Anxiety Knowledge (0-22)** | 9.5 (2.7) |  | 8.6 (3.2) |  |
| **Peer Emotional and Informational Social Support (0-100)** | 59.1 (25.3) |  | 60.2 (26.0) |  |
| **Parent-Adolescent Communication Quality (20-100)** | 61.2 (6.1) |  | 58.9±6.2) |  |
| Openness of Communication (10-50) | 30.1 (9.0) |  | 29.4 (10.2) |  |
| Extent of Communication (10-50) | 31.1 (8.2) |  | 29.4 (7.5) |  |
| **Parent-Adolescent Relationship Quality (1-5)** | 3.7 (1.1) |  | 3.7 (1.1) |  |
| **Perceived Need for Treatment** |  | 15 (75%) |  | 14 (78%) |
| **General-practice Users Perceived Need Inventory – Would like to discuss or already getting help for…** |  |  |  |  |
| Information about emotional problems |  | 13 (65%) |  | 14 (78%) |
| Medication |  | 10 (50%) |  | 9 (50%) |
| Counseling |  | 13 (65%) |  | 11 (61%) |
| Reports stopped from getting help due to: |  |  |  |  |
| Preference for self-management |  | 8 (40%) |  | 13 (72%) |
| Didn’t think anything would help |  | 3 (15%) |  | 5 (28%) |
| Didn’t know where to get help |  | 3 (15%) |  | 6 (33%) |
| Afraid to ask for help/what others would  think |  | 5 (25%) |  | 6 (33%) |
| Cost |  | 1 (5%) |  | 4 (22%) |
| Asked but didn’t get help |  | 1 (5%) |  | 2 (11%) |
| **Depressive Symptoms (0-27)** | 11.8 (5.5) |  | 10.8 (3.5) |  |
| **Anxiety Symptoms (0-21)** | 11.0 (5.8) |  | 8.9 (4.2) |  |
| **Adolescent Functioning** |  |  |  |  |
| General functioning (0-40) | 26.6 (5.8) |  | 23.2 (7.2) |  |
| Family functioning (0-28) | 18.9 (4.9) |  | 19.1 (5.4) |  |
| Peer functioning (0-24) | 17.5 (5.6) |  | 16.3 (3.0) |  |
| **Initial Treatment Recommendation** |  |  |  |  |
| Working on self-care such as sleep |  | 2 (10%) |  | 2 (11%) |
| Following up with the AHCP and/or PCP |  | 7 (35%) |  | 7 (39%) |
| Seeing a mental health professional to talk to |  | 18 (90%) |  | 12 (67%) |
| Starting a new medication for depression  or anxiety |  | 4 (20%) |  | 2 (11%) |

**Supplemental Table 2** **Full Baseline Characteristics for the Parent Study Sample**

| Baseline Characteristic | EUC | | SOVA | |
| --- | --- | --- | --- | --- |
|  | (n=13) | | (n=13) | |
|  | Mean (SEM) | N (%) | Mean (SEM) | N (%) |
| **Age** | 47.2 (5.2) |  | 43.8 ± (6.1) |  |
| **Gender Identity** |  |  |  |  |
| Female |  | 13 (100%) |  | 13 (100%) |
| **Race** |  |  |  |  |
| White |  | 11 (85%) |  | 13 (100%) |
| Black or African American |  | 2 (15%) |  | 0 (0%) |
| **Ethnicity** |  |  |  |  |
| Hispanic or Latino |  | 1 (8%) |  | 0 (0%) |
| **Education (highest level of schooling)** |  |  |  |  |
| High school graduate, diploma or  equivalent (for example: GED) |  | 1 (8%) |  | 3 (23%) |
| Some college credit, no degree |  | 2 (15%) |  | 3 (23%) |
| Trade/technical/vocational training |  | 2 (15%) |  | 2 (15%) |
| Associate degree |  | 1 (8%) |  | 1 (8%) |
| Bachelors degree |  | 3 (23%) |  | 4 (31%) |
| Masters degree |  | 3 (23%) |  | 0 (0%) |
| Professional degree (M.D., J.D.) |  | 1 (8%) |  | 0 (0%) |
| **Access to Transportation** |  | 11 (85%) |  | 9 (69%) |
| **Social Status Ladder – Community (1-10)** | 6.4 (1.9) |  | 5.5 (2.2) |  |
| **Social Status Ladder – United States (1-10)** | 6.0 (1.8) |  | 4.9 (2.2) |  |
| **Prior History of Child Receiving Psychotherapy^a^** |  | 11 (85%) |  | 5 (38%) |
| **Prior History of Child Receiving Medication^a^** |  | 3 (23%) |  | 2 (15%) |
| **Prior History of Parent Receiving Psychotherapy** |  | 9 (69%) |  | 10 (77%) |
| Number of Visits with Professional if  any | 21.8 (14.6) |  | 34.1 (37.7) |  |
| How helpful prior visits were (1-5) | 2.0 (1.5) |  | 3.3 (0.8) |  |
| **Prior History of Parent Receiving Medication** |  | 7 (54%) |  | 9 (69%) |
| How helpful medication was (1-5) | 2.7 (1.6) |  | 2.8 (1.2) |  |
| **Stigma – total (0-72)** | 21.0 (10.0) |  | 22.0 (10.8) |  |
| Personal Stigma (0-36) | 4.3 (4.5) |  | 6.2 (5.2) |  |
| Perceived Stigma (0-36) | 16.7 (9.2) |  | 15.9 (8.4) |  |
| **Acceptance of Antidepressant Use for Child (0-18)** | 13.5 (4.6) |  | 11.9 (6.1) |  |
| **Worry about Antidepressant Use for Child (0-24)** | 10.7 (8.8) |  | 11.8 (7.5) |  |
| **Parental Barriers to Adolescent Seeking Help from a Therapist (11-66)** | 38.6 (5.8) |  | 31.4 (11.4) |  |
| **Depression Knowledge (0-22)** | 14.9 (4.9) |  | 13.2 (3.1) |  |
| **Anxiety Knowledge (0-22)** | 11.2 (3.8) |  | 7.9 (4.3) |  |
| **Peer Emotional and Informational Social Support (0-100)** | 74.5 (18.3) |  | 60.1 (25.6) |  |
| **Parent-Adolescent Communication Quality (20-100)** | 60.4 (5.2) |  | 61.5 (5.7) |  |
| Openness of Communication | 39.1 (6.4) |  | 37.1 (7.0) |  |
| Extent of Communication | 21.3 (5.3) |  | 24.5 (6.8) |  |
| **Parent-Adolescent Relationship Quality (1-5)** | 4.3 (0.5) |  | 3.9 (0.8) |  |
| **Perceived Need for Treatment for Child** |  | 13 (100%) |  | 10 (77%) |
| **General-practice Users Perceived Need Inventory – Would like to discuss or already getting help for your child…** |  |  |  |  |
| Information about emotional problems |  | 13 (100%) |  | 10 (77%) |
| Medication |  | 10 (77%) |  | 7 (54%) |
| Counseling |  | 13 (100%) |  | 10 (77%) |
| Reports stopped from getting help due to: |  |  |  |  |
| Preference for parent to manage  child’s emotional problems by  themselves |  | 0 (0%) |  | 0 (0%) |
| Didn’t think anything would help  child |  | 1 (8%) |  | 0 (0%) |
| Didn’t know where to get help for  child |  | 1 (8%) |  | 3 (23%) |
| Afraid to ask for help/what others  would think of them as a parent |  | 0 (0%) |  | 1 (8%) |
| Cost |  | 2 (15%) |  | 2 (15%) |
| Asked but didn’t get help for child |  | 0 (0%) |  | 0 (0%) |
| **Adolescent Functioning (Columbia Impairment Scale) 0-52** | 14.8 (8.1) |  | 14.5 (7.6) |  |
